# DAVID Knowledgebase: a gene-centered database integrating heterogeneous gene annotation resources to facilitate high-throughput gene functional analysis

**DOI:** 10.1186/1471-2105-8-426

**Published:** 2007-11-02

**Authors:** Brad T Sherman, Da Wei Huang, Qina Tan, Yongjian Guo, Stephan Bour, David Liu, Robert Stephens, Michael W Baseler, H Clifford Lane, Richard A Lempicki

**Affiliations:** 1 Laboratory of Immunopathogenesis and Bioinformatics, Clinical Services Program, SAIC-Frederick, Inc., National Cancer Institute at Frederick, Frederick, MD 21702, USA; 2 Laboratory of Immunoregulation, National Institute of Allergy and Infectious Diseases, National Institutes of Health, Bethesda, MD 20892, USA; 3 Advanced Biomedical Computing Center, SAIC-Frederick, Inc., National Cancer Institute at Frederick, Frederick, MD 21702, USA; 4 Bioinformatics and Scientific IT Program, NIAID Office of Technology Information Systems, National Institute of Allergy and Infectious Diseases, National Institutes of Health, Bethesda, MD 20892, USA; 5 Clinical Services Program, SAIC-Frederick, Inc., National Cancer Institute at Frederick, Frederick, MD 21702, USA

## Abstract

**Background:**

Due to the complex and distributed nature of biological research, our current biological knowledge is spread over many redundant annotation databases maintained by many independent groups. Analysts usually need to visit many of these bioinformatics databases in order to integrate comprehensive annotation information for their genes, which becomes one of the bottlenecks, particularly for the analytic task associated with a large gene list. Thus, a highly centralized and ready-to-use gene-annotation knowledgebase is in demand for high throughput gene functional analysis.

**Description:**

The DAVID Knowledgebase is built around the DAVID Gene Concept, a single-linkage method to agglomerate tens of millions of gene/protein identifiers from a variety of public genomic resources into DAVID gene clusters. The grouping of such identifiers improves the cross-reference capability, particularly across NCBI and UniProt systems, enabling more than 40 publicly available functional annotation sources to be comprehensively integrated and centralized by the DAVID gene clusters. The simple, pair-wise, text format files which make up the DAVID Knowledgebase are freely downloadable for various data analysis uses. In addition, a well organized web interface allows users to query different types of heterogeneous annotations in a high-throughput manner.

**Conclusion:**

The DAVID Knowledgebase is designed to facilitate high throughput gene functional analysis. For a given gene list, it not only provides the quick accessibility to a wide range of heterogeneous annotation data in a centralized location, but also enriches the level of biological information for an individual gene. Moreover, the entire DAVID Knowledgebase is freely downloadable or searchable at .

## Background

In the post-genomic era, one of the challenges is to systematically and comprehensively interpret large amounts of data results from experiments with a genome-wide scope, such as gene lists derived from microarray or proteomics studies. Using the biological knowledge accumulated in the past decades and the aid of computing algorithms, it is possible to assemble potential biological pictures associated with these studies. Due to the complex and distributed nature of biological research, our current knowledge is spread over many redundant databases maintained by independent groups. One gene could have different identifiers within one, or many, databases. Similarly, the biological terms associated with different gene identifiers for the same gene could be collected in different levels across different databases. Thus, an integrated gene-annotation database with comprehensive data coverage is essential as the first step of any high-throughput gene functional analytic algorithm. Some integrated databases, such as NCBI Entrez Gene [1], UniProt [2], PIR [3], etc., made great efforts to integrate annotation resources in one centralized location and are considered to be the world-class bioinformatics foundation for general bioinformatics purposes. A couple of other projects, e.g. SOURCE [4], RESOURCER [5], IDconverter [6], BioMart (formerly EnsMart) [7], UCSC Gene Sorter [8], were developed towards being more suitable for high throughput gene-annotation queries. However, some areas are still needed for further developments in order to better meet the requirements of the high throughput gene analysis: 1) Many types of annotations are not included. e.g. Panther and BioCarta Pathways are not covered in any of above works. 2) The partial cross-reference between NCBI and UniProt systems limits integration capability. e.g. Entrez Gene does not cover PIR ID or Affy ID at all. 3) The resulting format could be better suitable for high throughput data analysis of multiple genes. 4) The web query is performed on one gene at a time or in a small batch mode. e.g. only 100 gene at-at-time in Entrez Gene. 5) The database download is too large and complicated for regular users. e.g. Entrez Gene is in the range of tens of gigabytes in size and is comprised of a complicated, xml-like structure. 6) All data for a given database is not always available. e.g. SOURCE does not offer downloads. Due to the above limitations, the scope of most high-throughput functional annotation algorithms or data analyses is limited to a small subset of the many annotation resources and ID systems available, which does not maximize the potential analytic power. For example, the gene-annotation enrichment analytic tools, e.g. GOMiner [9], ermineJ [10], GOStat [11], etc., only use the GO database [12] as a backend annotation source and only NCBI Entrez Gene as a gene ID mapping source. Gene IDs and annotation contents derived from Uniprot are weaker or not acceptable at all in these packages. In addition, each of the tools requires a large amount of redundant efforts to build its own backend database from public resources.

The goal of this work is to create a large gene-centered knowledgebase that integrates the most useful and highly regarded heterogeneous annotation resources in a centralized location with improved cross-referencing capability between NCBI and UniProt systems [1,2], and easy to use pair-wise data structure files for downloads, hence, more comprehensive and suitable for high throughput data analysis. The work was originally conducted years ago to successfully serve as a comprehensive backend knowledgebase for various high throughput gene-annotation enrichment analytic tools in the DAVID and EASE packages [13,14]. The usefulness of the DAVID knowledgebase in our own bioinformatics software motivates us to make it available to the public community in order to benefit the high throughput data analysis projects in other research groups. Now, the entire DAVID Knowledgebase [15] is either freely downloadable or searchable through the DAVID Bioinformatics Resources web site [16].

The paper will describe the DAVID Knowledgebase regarding its unique strategy to integrate the redundant and heterogeneous annotation sources, the improved cross-reference capability across gene ID types, the large annotation content coverage, and the pair-wise text-format files for downloads and easy-to-use web-based query interface.

## Construction and content

**Figure 1:**
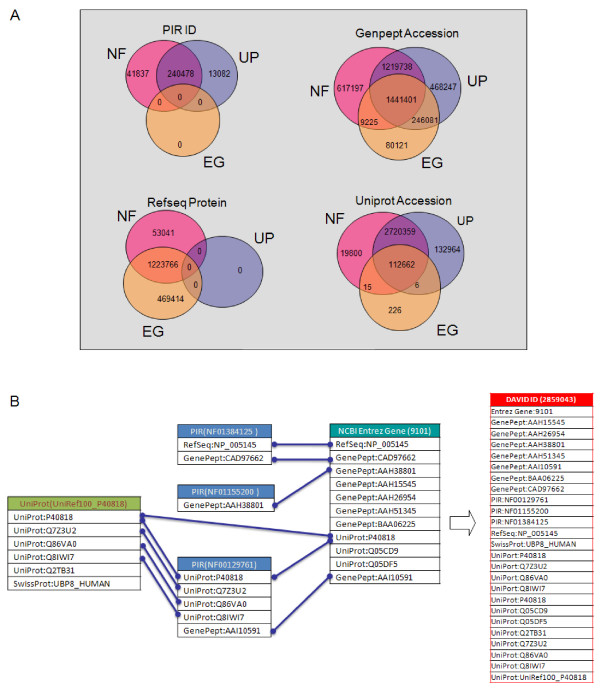
The cross-reference among different types of gene identifiers is improved by the DAVID Gene Concept. A. As global examples, four popular types of protein identifiers (PIR ID, UniProt Accession, RefSeq Protein, and GenPept Accession) are only cross-referenced partially by NCBI Entrez Gene (EG), UniProt UniRef100 (UP), and PIR NRef100 (NF). B. The DAVID Gene Concept, a single-linkage algorithm, iteratively agglomerates all types of gene IDs, belonging to the same gene entry, for example, *ubiquitin specific peptidase 8 (USP8)*, into one DAVID gene cluster (ID 2859041). Such integration makes the cross-reference of different types of gene IDs more comprehensive than that in each of the original databases, particularly for the IDs across NCBI and UniProt systems.

**Figure 2:**
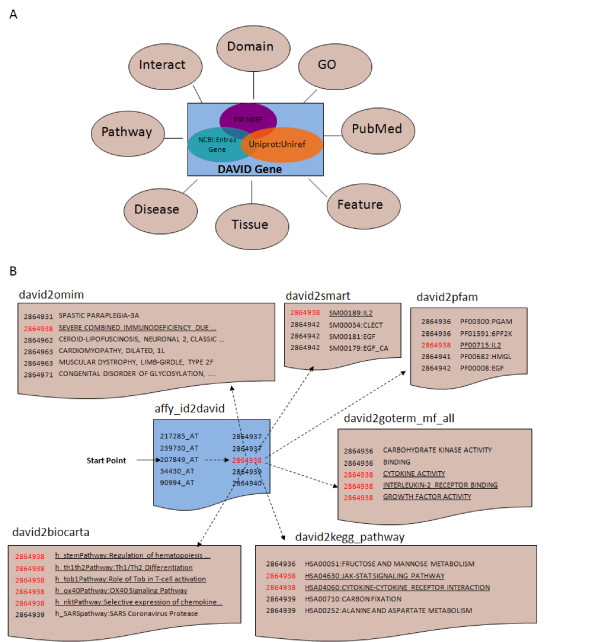
The gene-centric DAVID Knowledgebase in a simple pair-wise text format centralized by DAVID gene identifiers. A. A hypothetical graph shows that a wide-range of annotation categories are collected and integrated by the DAVID gene cluster IDs. B. A real example demonstrates how the pair-wise text data structures are used in the DAVID Knowledgebase. Each independent annotation source or gene identifier system is separated into independent files in the same pair-wise format of "DID-to-annotation". In this example, a user starts with an Affymetrix identifier (affy_id) 207849_at (IL2). The first step is to obtain the corresponding DAVID gene identifier (DID:2864938). Then, with this DID (red), the annotation terms of interest (underlined) in different source files (OMIM, SMART, Pfam, GO Molecular Function, KEGG Pathway, BioCart Pathway, etc.) can be queried sequentially.

Most gene functional annotation databases are in a gene-annotation association format, i.e. annotation contents usually associate with corresponding gene or protein identifiers. Such a format provides an opportunity to integrate heterogeneous annotation resources through their common gene identifiers. The construction of the DAVID Knowledgebase consists of two major steps: 1) Improve cross-reference capability across redundant gene/protein IDs, particularly for the IDs found in the NCBI and UniProt systems [1,2], with a novel single-linkage algorithm called the DAVID Gene Concept. As a result, unique DAVID gene clusters are formed to hold the redundant gene/protein IDs belonging to the same gene entries (Figure 1B). 2) Assign heterogeneous annotation contents from different annotation databases, which could be associated with different types of gene/protein IDs (but belonging to the same gene), to the same DAVID gene cluster (Figure 2).

### DAVID gene concept: a novel single-linkage algorithm to agglomerate redundant gene IDs into the DAVID gene clusters in order to improve cross-referencing capability

There are dozens of types of gene or protein sequence identifiers that are redundant within the same group or across several independent groups, such as GenBank Accession; GenBank ID; RefSeq Accession; PIR ID; PIR Accession; UniProt ID; UniProt Accession; etc. [1-3,17-19]. The leading organizations, NCBI [1] and UniProt [2], have made significant strides in addressing the cross-reference and redundancy issues associated with gene identifiers. NCBI GenBank, representing the largest redundant database of nucleotide sequences, exchanges data with two other worldwide nucleotide sequence databases, EMBL and DDBJ. In addition, UniProt, as the largest redundant annotated protein sequence database, unites Swiss-Prot, TrEMBL, and PIR. Moreover, the three organizations have been independently constructing non-redundant gene cluster databases, NCBI Entrez Gene [1], UniProt UniRef [2], and PIR-NREF [3], respectively. The resulting databases are presented in a non-redundant format by grouping the different gene/protein IDs for the same gene into one entry. At this point, the redundant nucleotide and protein IDs from different resources have been largely addressed by the leading bioinformatics organizations. However, while the gene clusters are comprehensive for the gene/protein IDs within their own organization, many cannot be cross-referenced with gene identifiers from other independent organizations (Figure 1A). For example, UniProt does not cover RefSeq IDs; NCBI Entrez Gene does not reference PIR ID at all (Figure 1A). Therefore, the major challenge of annotation integration comes from the weak cross-reference of different types of gene/protein IDs between NCBI and UniProt systems since different annotation databases use one or another system as their major gene identifier systems, e.g. GeneRif [20] adopts NCBI IDs as major associated identifiers; InterPro [21] uses UniProt/SwissProt as major associated identifiers.

To further improve the cross-reference capability among different types of gene/protein identifiers, DAVID gene clusters, as secondary gene clusters, are created by merging the existing gene clusters from three major gene cluster databases, Entrez Gene [1], UniRef100 [19], and PIR-NREF100 [3], with a single-linkage algorithm (Figure 1B). Any gene clusters from the above three resources with one or more protein IDs in common and from the same species will be considered as the same gene entry. The overlapping gene clusters are iteratively merged into a new gene cluster based on the single-linkage rule until all final gene clusters, or DAVID genes, are stable (Figure 1B). A unique integer number is assigned to each of the newly formed DAVID genes and is used as a centralized gene identifier/index within the DAVID Knowledgebase (Figure 1B). Importantly, the DAVID gene ID is a gene level clustering ID so that any different isoforms or splicing variants belonging to the same gene will be assigned to the same DAVID ID. The process collects >60 million individual gene/protein identifiers representing over 20 identifier types (Table 1), which are eventually agglomerated into over 3.7 million DAVID genes, for over 90,000 species. The DAVID genes greatly improve the cross-referencing capability for the IDs found in the NCBI and UniProt systems.

**Table 1:** Data coverage in the DAVID Knowledgebase.

**Gene Identifiers (> 60 millions)**	**Annotation Contents (> 90 millions in total)**		
AFFY_ID	**Ontology (>40 million records)**	**Domain/Family (> 15 millions)**	**General Annotation (>21 millions)**
ENTREZ_GENE_ID	GO_BIOLOGICAL PROCESS	BLOCKS_ID	ALIAS_GENE_SYMBOL
GENPEPT_ACCESSION	GO_MOLECULAR FUNCTION	COG_KOG_NAME	CHROMOSOME
GENBANK_ACCESSION	GO_CELLULAR COMPONENT	INTERPRO_NAME	CYTOBAND
GI	PANTHER_BIOLOGICAL PROCESS	PDB_ID	GENE_NAME
PIR_ACCESSION	PANTHER_MOLECULAR FUNCTION	PFAM_NAME	GENE_SYMBOL
PIR_ID	COG_KOG_ONTOLOGY	PIR_ALN	HOMOLOGOUS_GENE
PIR_NREF_ID	**P-P Interaction (> 4 millions)**	PIR_HOMOLOGY_DOMAIN	LL_SUMMARY
REFSEQ_GENOMIC	BIND	PIR_SUPERFAMILY_NAME	OMIM_ID
REFSEQ_MRNA	DIP	PRINTS_NAME	PIR_SUMMARY
REFSEQ_PROTEIN	MINT	PRODOM_NAME	PROTEIN_MW
REFSEQ_RNA	NCICB_CAPATHWAY	PROSITE_NAME	REFSEQ_PRODUCT
UNIGENE	TRANSFAC_ID	SCOP_ID	SEQUENCE_LENGTH
UNIPROT_ACCESSION	HIV_INTERACTION	SMART_NAME	SP_COMMENT
UNIPROT_ID	HIV_INTERACTION_CATEGORY	TIGRFAMS_NAME	**Functional Category (>6.9 millions)**
UNIREF100_ID	HPRD_INTERACTION	PANTHER_SUBFAMILY	PIR_SEQ_FEATURE
OFFICIAL_GENE_SYMBOL	REACTOME_INTERACTION	PANTHER_FAMILY	SP_COMMENT_TYPE
ESSENBLE_ID	**Disease Association (~9,000)**	**Pathways (>50,000)**	SP_PIR_KEYWORDS
FLYBASE_ID	GENETIC_ASSOCIATION_DB	BioCarta	UP_SEQ_FEATURE
HAMAP_ID	OMIM_PHENOTYPE	KEGG_PATHWAY	**Gene Expression (>1.0 million)**
HSSP_ID	**Literature (>2.8 millions)**	PANTHER_PATHWAY	GNF Microarray
TIGR_ID	GENERIF_SUMMARY	PID	UNIGENE EST
WORMBASE_ID	PUBMED_ID	BBID	CGAP SAGE
RGD_ID	HIV_INTERACTION_PUBMED_ID	KEGG_REACTION	CGAP EST
UNIPROT_ACCESSION			

Comprehensive collection and integration of different types of gene identifiers and heterogeneous annotation categories. Importantly, any of the gene identifier types and associated annotation categories above are comprehensively cross-mapped to each other in the DAVID Knowledgebase by the unique DAVID Gene Concept procedure.

Because a DAVID gene is built based on annotated gene clusters, only well-known or studied gene identifiers in the original gene clusters are included. This scope is well aligned with the high throughput functional annotation purpose of the DAVID Knowledgebase in the sense that any unclear or unstudied sequences, such as an EST, are not helpful for automatic analysis of high-throughput functional annotation.

DAVID genes are secondary gene clusters built on well-known and annotated gene clusters from NCBI Entrez Gene, PIR NRef100, and UniProt UniRef100. Thus, the agglomeration quality of a DAVID gene solely relies on the quality of the original databases. Since the original databases have been used by the scientific community for many years, they are well known and regarded as the highest-quality bioinformatics resources in the world. To further detect potential problems inherited from the original sources into DAVID gene clusters, a comprehensive quality control (QC) procedure was conducted by examining the sequence alignment of every protein member within a given DAVID gene cluster using the NCBI BlastClust program [22,23] (Additional File 1 for detailed procedure of QC). The QC examination highlighted poor sequence alignment in ~10% of the DAVID genes, mainly caused by very short sequences (less than 20 amino acids), which were not handled well, or at all, by the BlastClust program. After filtering out those short sequences, less than 0.1% of the DAVID genes with poor alignment members needed to be corrected. The QC procedure reflects the high quality of the original resources, which is passed on to the DAVID Knowledgebase.

### Collection and integration of functional annotation contents: the heterogeneous annotation contents and their IDs from different annotation databases are assigned to and centralized by the common DAVID genes

**Figure 3:**
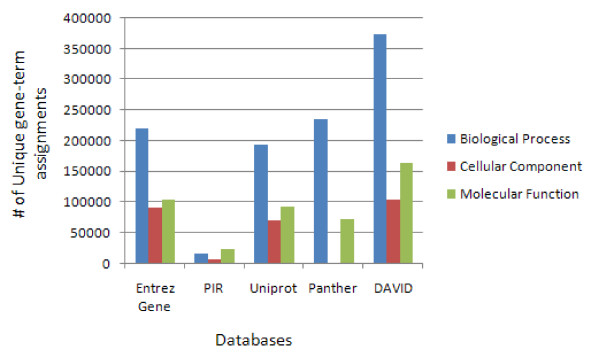
The improved annotation coverage in the DAVID Knowledgebase. For all genes in the entire human genome, the coverage of Gene Ontology annotations, one of the most highly used annotation resources, is 10–20% greater in the DAVID Knowledgebase than that in other individual source (e.g. Entrez Gene, PIR, Panther and UniProt).

NCBI Entrez Gene, PIR, and UniProt databases [1-3] also collect annotation contents associated with corresponding gene IDs. But the integration is dependent on the gene ID system, i.e. one database may not fully integrate some annotation contents that are associated with gene identifiers not well cross-referenced within its system. For instance, InterPro associated with SwissProt/UniProt IDs may not be comprehensively integrated into the NCBI system. However, the situation is improved with the larger collection and higher integration of different types of gene identifiers within the DAVID genes (Table 2). Since all major types of gene identifiers eventually can be translated to a corresponding DAVID gene, the heterogeneous annotation contents, as long as they are in a gene-annotation association format, have a much better chance of being integrated by the common DAVID gene, hence improving the integration of annotation contents for individual gene (Table 2), as well as for entire genome as a whole (Figure 3). The DAVID Knowledgebase collects a wide range of well-known and high-quality annotation contents from dozens of databases including: Gene Ontology; Protein Domains; Bio-pathways; Gene Expression; Disease Association; PubMed; Protein-Protein interactions; Affymetrix; Gene General Features; NCI Thesaurus; Panther Family; and more (Table 1) (also see Additional File 2 for the annotation coverage comparison across databases). Furthermore, the structure of the DAVID Knowledgebase is open to any new annotation database as long as it is in a gene-annotation association format (Figure 2).

**Table 2:** An example of improved annotation coverage for an individual gene *USP8 *in the DAVID Knowledgebase.

**Category (Resource)**	**Selected Annotation**	**Entrez Gene**	**UniProt**	**PIR iProClass**	**DAVID**
Molecular Function (Gene Ontology)	GO:0004197:cysteine-type endopeptidase activity	√	√	√	√
	GO:0005515:protein binding	√	√	√	√
	GO:0004843:ubiquitin-specific protease activity	√	√	√	√
	GO:0008233: peptidase activity			√	√
	GO:0004221: ubiquitin thiolesterase activity			√	√
	GO:0016787: hydrolase activity			√	√
	GO:0008234: cysteine-type peptidase activity			√	√
Biological Process (Gene Ontology)	GO:0006512: ubiquitin cycle	√	√	√	√
	GO:0008283: cell proliferation	√	√	√	√
	GO:0006511: ubiquitin-dependent protein catabolic process	√	√	√	√
	GO:0007265: Ras protein signal transduction			√	√
Cellular Component (Gene Ontology)	GO:0005622: intracellular			√	√
Protein Domain (InterPro)	IPR001394:Peptidase_C19		√		√
	IPR001763:Rhodanese-like		√		√
Enzyme (EC)	EC 3.1.2.15: Ubiquitin thiolesterase		√		√
Protein 3-D Structure(PDB)	1WHB:Solution structure of the Rhodanese-like domain in human UBP8			√	√
	2A9U: Structure of the N-terminal domain of HumanUSP8			√	√
Disease Association (OMIM)	OMIM:603158: chronic myeloproliferative disorder	√	√		√

For the gene of *USP8*, the annotation coverage is partially overlapped among the independent resources of Entrez Gene, UniProt and PIR. After the integration around DAVID Gene Concept (Figure 1B), the DAVID Knowledgebase provides more comprehensive coverage of annotation in one centralized location for *USP8 *than that in each of the individual sources.

Inaccurate and conflicting annotation has been considered due to its potential negative impact on the knowledgebase. This impact is taken into consideration and corrected during DAVID cluster creation within the DAVID Quality control pipeline as stated previously. While the incorrect annotation is not used in the creation of the DAVID clusters, thereby stopping any magnification of error, the annotation is still maintained in the knowledgebase. Due to the significant amount of data available within the knowledgebase, including redundant and complimentary annotation, the inaccurate data becomes highly diluted. Considering that the DAVID Knowledgebase is intended for high throughput gene functional analysis, when many biology aspects are considered together in either high-throughput analysis or for any one gene, the negative impact of any errors is negligible since the true biology is generally overwhelming and supporting from the various sources. This may not be the case with each individual database if there is not a majority of supporting evidence given for the true biology or a systematic error has occurred with the source's annotation process for a given gene.

Considering the DAVID Knowledgebase is designed for high throughput gene functional analysis, the larger collection and integration of heterogeneous annotation sources and the quick accessibility to the larger amount of data are more important than the timely update, simply because the high throughput gene functional analysis relies on the global annotation profiles rather than an individual annotation source. Through automation of several tasks where appropriate and personnel additions, the goal of the DAVID knowledgebase update is set to occur quarterly. A complete list of the public databases contributing to the DAVID Knowledgebase are provided within Addiitonal File 3 while a detailed update procedure can be found in Additional File 4.

## Utility and discussion

### The data structure of the entire knowledgebase for downloads

The DAVID Knowledgebase is available in two categories of pair-wise text files: gene index files (gene id knowledge) and annotation index files (annotation knowledge) [15]. Gene index files with a naming convention such as david2affy_id, david2genbank_accession, etc., are in a pair-wise format, linking a DAVID gene identifier to a public gene identifier (Figure 2). These relationships were built based on the results from the DAVID gene agglomeration step described in the "Construction and Content" section. Thus, any given public gene identifier can be converted to a corresponding DAVID gene identifier that represents a unique gene entry and internal linker to all available annotation contents within the DAVID Knowledgebase. Annotation index files with a naming convention such as david2pfam, david2kegg_pathway, david2omim, etc., are also in a format that pairs a DAVID gene identifier with an annotation term. All genes and annotation terms within the DAVID Knowledgebase are centralized by the common DAVID gene identifier. The unified DAVID gene identifier not only normalizes the cross-reference among heterogeneous databases, but also makes any search and calculation simpler and more efficient. Therefore, for any given public gene identifier, the corresponding DAVID gene identifier can be obtained with the DAVID gene index files. Then, any annotation terms within the DAVID Knowledgebase can be further queried from the DAVID annotation index files using the DAVID identifier (Figure 2B). In addition, each independent annotation resource and public gene identifier system is separated into independent files, making it easier for users to focus on the data they are most interested in, as well as reducing the file size and simplifying the format for easy processing. The easily interpreted file names, such as david2genbank_accession and david2pfam, allow users to quickly identify the data that will help them interpret their data. Of course, users can further combine their data files of interest into one large file or database table to search the data in a way best suited to the individual. The simple pair-wise text format provides the flexibility to either directly query the files or insert them into tables in a customized, in-house relational database without much, if any, file parsing or re-formatting. The format should be simple to regular users with some computational skills as well as extendable for expert users. Moreover, users may add any new annotation sources to the DAVID Knowledgebase as additional independent files, as long as the files are in a gene-annotation associated format. The DAVID Knowledgebase, with its large, diverse annotation categories and flexible format, provides the scientific community with a single, comprehensive platform for gathering annotation for specific studies.

### Web interface for batch query

**Figure 4:**
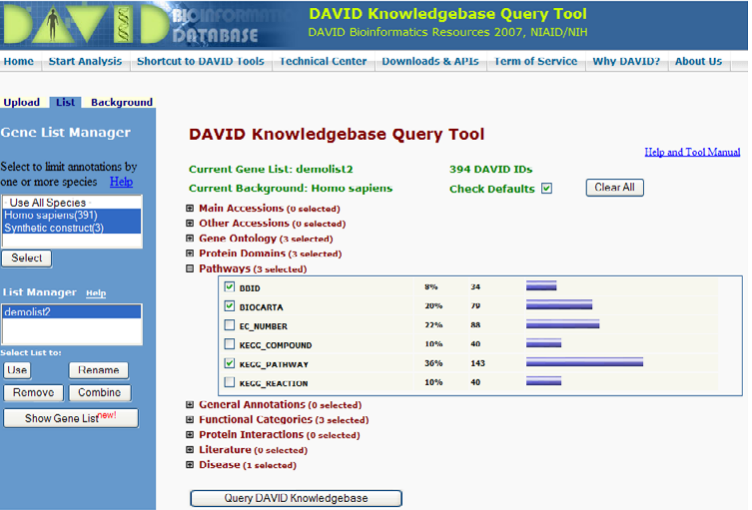
The web-based interface of the DAVID Knowledgebase to query annotation for given gene list. Firstly, users can submit various types of gene identifiers through the 'Gene List Manager' panel on the left side. Then the associated annotation categories may be selected and queried through the right panel accordingly.

In addition to the database download for genome-wide large scale analysis, the DAVID Knowledgebase can be queried in a gene-centric way (i.e. from gene to annotation) for small (e.g. a couple of hundreds) to medium size (e.g. a couple of thousands) of gene lists through an easy-to-use web interface [24] (Figure 4). The annotation contents from different databases on the web site interface are organized into 10 groups such as Pathways, Protein-Protein Interaction, Gene Ontology, etc. which organization is consistent with that in the entire database downloads (Table 1). Flexible options are provided in order for users to define the query scope of the annotation categories which relate to their interests. After a gene list is submitted, the web interface efficiently queries the chosen annotation contents for gene/protein IDs in the given list (Figure 4, also see Additional File 5). The query results for all genes are organized in one single page/file, which is more suitable for high throughput data analysis. Then, users may either further explore the annotation contents on the html report or download the gene-annotation results in a flat text file. In addition, for light duty jobs, a web-based API service [25] is also available for users to query the DAVID Knowledgebase through a URL link on a third party web site or an automatic script.

### A query comparison between DAVID Knowledgebase and Entrez Gene

**Figure 5:**
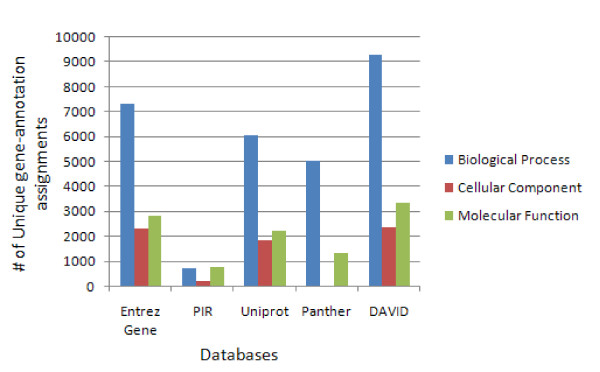
About 10–20% increase of gene-GO annotation assignments for the ~400 Affymetrix IDs (additional file 4) derived from a HIV microarray study [26].

Due to the comprehensive integration procedure, the DAVID Knowledgebase provides not only quick access to a wide range of annotation contents in one location but also enriched annotation contents and improved ID cross-referencing capability. To the best of our knowledge, these features exceed that of other similar integrated sources. A list of 400 Affymetrix IDs (see Additional File 6)  derived from a HIV microarray study (labeled as demolist 2 on the DAVID web site) [26] is used to query the DAVID Knowledgebase and NCBI Entrez Gene Database respectively in order to obtain associated biological annotations. The first issue arises due to the fact that the Entrez Gene database does not recognize Affymetrix IDs. Thus, we have to convert the Affymetrix IDs to their corresponding Entrez Gene IDs in order to take advantage of the annotation contents in the Entrez Gene database. In contrast, DAVID can directly recognize Affymetrix IDs and most other types of IDs. Secondly, since the Entrez Gene database is designed for general bioinformatics purposes (maximally 100 gene IDs at-a-time), the 400 IDs have to be split into a couple of smaller batches in order to perform the queries. Then, the results have to be manually merged and combined. Conversely, DAVID Knowledgebase supports high throughput access, and the results are put into one single file. Thirdly, the heterogeneous annotation contents in Entrez Gene Report appear in multiple rows, in many big blocks, across multiple genes. Conversely, the annotation contents in the DAVID download page are organized into one row for one gene where annotations are separated by tab delimitation. This organization can immediately be entered into MS Excel for further processing. Finally, the most important advantage of the DAVID Knowledgebase is that the annotation categories are much extended and the annotation contents are enriched. For the example Affymetrix list, 10–20% more GO terms are enriched in the DAVID Knowledgebase compared to each of the individual resources (e.g. Entrez Gene) (Figure 5). A similar conclusion was obtained when we conducted comparisons to SOURCE [4], RESOURCER [5], IDconverter [6], BioMart [7], and UCSC Gene Sorter [8] with the same gene list [26] (see Additional File 7 for detailed results).

### Key advantages of the DAVID Knowledgebase

The DAVID Knowledgebase intends to integrate and organize the high quality, world-class bioinformatics databases into a centralized location in a gene-centric format. The work is particularly useful for high throughput data analysis with the following advantages: 1) Improved ID cross-referencing capability enhances comprehensiveness of integration of heterogeneous annotation resources, hence enriching the annotation coverage for individual genes. 2) The integration allows quick access to a wide range of annotation contents in a batch manner. 3) Simple pair-wise, gene-centric formatted files simplify the data structure so that all users may benefit. 4) The pair-wise data structure is more flexible and suitable for high throughput data access.

### Special attention on the DAVID Knowledgebase

After several QC procedures, a certain error rate still exists in the DAVID Knowledgebase as it exists in any other bio-databases. Users may report such errors directly to us through email or the DAVID forum [27]. In addition, users should be aware that the DAVID Knowledgebase is designed for high throughput gene functional screening for large gene lists on a gene-centric level, rather than replacing original annotation databases, which may contain additional details, such as gene isoform specific annotations, for drill-down analysis. Moreover, the quarterly update schedule of the DAVID Knowledgebase could result in a slight time gap as compared with the member database updates.

## Conclusion

The DAVID Gene Concept agglomerates diverse types of gene identifiers belonging to the same gene into one gene cluster. It allows large collections of heterogeneous annotations that are associated with different types of gene identifiers to be comprehensively integrated by a common DAVID gene. Combined with the simple pair-wise text format, the DAVID Knowledgebase provides not only a comprehensive, high-quality collection of gene annotation resources, but also the flexibility to cross-reference identifiers and annotations from several world-class, heterogeneous databases within one resource. To the best of our knowledge, the annotation data coverage and gene/protein ID cross-referencing capability far exceeds that of backend data sources of other high throughput gene functional annotation tools. Therefore, it can be used as the backend gene-annotation database of existing high throughput gene functional analysis tools to improve their discovery power. The DAVID Knowledgebase also aids the researcher in focusing on data analysis or the core development of new high-throughput functional data-mining algorithms, rather than spending time on gene-annotation data collection and integration.

## Availability and requirements

The DAVID Knowledgebase is freely downloadable for nonprofit use under the URL . Data files are available in a tab-delimited text format which can be opened by any text editor in Windows, Mac or Unix systems.

## Abbreviations

DAVID: Database for Annotation, Visualization and Integrated Discovery

PIR: Protein Information Resource

NCBI: National Center for Biotechnology Information

BLAST: Basic Local Alignment Search Tool

EMBL: European Molecular Biology Laboratory

DDBJ: DNA Data Bank of Japan

RefSeq: Reference Sequence

GO: Gene Ontology

SMART: Simple Modular Architecture Research Tool

DID: DAVID Identifier

EG: Entrez Gene

UP: UniProt-UniRef

NF: PIR-NRef

OMIM: Online Mendelian Inheritance in Man

Pfam: Protein Family

LIB: Laboratory of Immunopathogenesis and Bioinformatics

## Authors' contributions

DWH oversaw the project and wrote the manuscript; BS developed the majority of the JAVA programs used to cluster the DAVID genes, and to parse and integrate annotation data; built the database architecture; and revised the manuscript. DWH and QT developed some programs contributing to additional annotation data or web interface; YG and SB developed the QC pipeline for the DAVID gene. JK developed some Perl scripts to add additional annotation data. DL and RS supported the Oracle database. RL and CL are principle investigators, who supervised the project design and implementation, and the manuscript preparation. All authors read and approved the final manuscript.

## Supplementary Material

Additional file 1 Flow chart of the procedure for DAVID gene QC with NCBI BlastClust program. Click here for file

Additional file 2 The Cross Comparisons of Gene Identifers and Annotation Categories among some integration databases, i.e. NCBI Entrez Gene; UniProt; PIR; SOURCE; RESOURCER; BioMart; UCSC Gene Sorter and DAVID Knowledgebase. Different projects offer different web site query flow; data file structures, and downloads. However, the comprehensive coverage and integration of the heterogeneous annotation sources in one centralized place are the No. 1 priority of the DAVID Knowledgebase for the success of high throughput gene functional analysis. Thus, the comparisons across the databases are mainly focusing on the annotation categories that are covered by the DAVID Knowledgebase, but may not be covered by other works. Note: '1' represents the corresponding annotation category collected in the database; and '0' represents not. In addition, due to most, if not all, of the databases do not directly and clearly list the covering annotation categories, the survey is based on data presented on their web interfaces. Thus, the survey is just for the purpose of general comparison with certain inaccuracy. Click here for file

Additional file 3 A complete list of the public databases contributing to the DAVID Knowledgebase. Click here for file

Additional file 4 An outline of the update procedure for the DAVID Knowledgebase. Click here for file

Additional file 5 The performance of querying the DAVID Knowledgebase through the web interface with different size gene lists. Click here for file

Additional file 6 The list of Affymetrix IDs from a HIV microarray study [26] used in the annotation query comparisons across databases. Click here for file

Additional file 7 The comparisons of querying GO terms with DAVID, SOURCE, RESOURCER, IDConverter, BioMart and UCSC Gene Sorter for the demolist 2 [26]. Click here for file
