# Supplementary material for: DAVID Knowledgebase: a gene-centered database integrating heterogeneous gene annotation resources to facilitate high-throughput gene functional analysis
Source: BMC Bioinformatics. 2007 Nov 2;8:426. doi: 10.1186/1471-2105-8-426 (PMC2186358; doi:10.1186/1471-2105-8-426)
Supplement: Additional file 1 — Flow chart of the procedure for DAVID gene QC with NCBI BlastClust program. [file 1471-2105-8-426-S1.doc]

DAVID Quality Control Pipeline


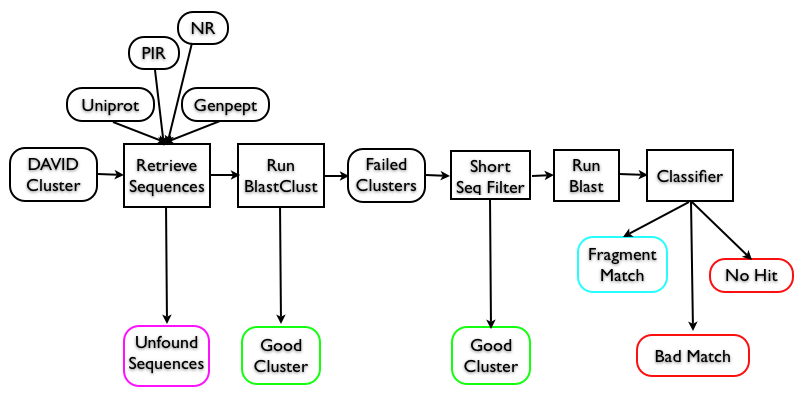


The protein sequences under each Human DAVID Gene are retrieved either from the local sequence blast databases (NR, Genpept, PIR, Swiss-Prot and TrEMBL) with FASTACMD embedded Blastall package, or from GenBank and Swiss-Prot online databases with in-house scripts developed in BioPerl. To examine the quality of each DAVID Gene cluster, the NCBI BlastClust program was run on the protein sequences with default thresholds. A short sequence filter (<=20 amino acids) was applied prior to each of the alignment jobs in order to minimize the major factor of the poor alignments. The NCBI Blast program was further used to distinguish the reasons for the poor alignments.
