# Supplementary material for: DAVID Knowledgebase: a gene-centered database integrating heterogeneous gene annotation resources to facilitate high-throughput gene functional analysis
Source: BMC Bioinformatics. 2007 Nov 2;8:426. doi: 10.1186/1471-2105-8-426 (PMC2186358; doi:10.1186/1471-2105-8-426)
Supplement: Additional file 3 — A complete list of the public databases contributing to the DAVID Knowledgebase. [file 1471-2105-8-426-S3.doc]

| **Contributions of Public Data Resources to DAVID Knowledge Base Construction**    **Listed by categories:** | | |
| --- | --- | --- |
| - **Primary Data Sources: Used for DAVID gene clustering** | | |
|  | - | NCBI Entrez Gene and LocusLink:  <http://www.ncbi.nlm.nih.gov/Entrez/> |
|  | - | UniProt and UniRef100: <http://www.pir.uniprot.org/> Swiss-Prot entries are copyrighted. They are produced through a collaboration between the Swiss Institute of Bioinformatics and the EMBL outstation - the European Bioinformatics Institute. There are no restrictions on their use by non-profit institutions as long as their content is in no way modified and this statement is not removed. Usage by and for commercial entities requires a license agreement (See http://www.isb-sib.ch/announce/ or send an email to license@isb-sib.ch) |
|  | - | PIR NREF and iProClass: <http://pir.georgetown.edu/> |
| - **Secondary Sources of Annotation: Used to map additional annotation to DAVID Genes** | | |
|  |  | **Affymetrix Probeset Mapping** - NetAffx: <http://www.netaffx.com/> - TIGR: <http://www.tigr.org/tdb/> |
|  |  | **Gene Ontology** **-** Data extract from [LocusLink](http://www.ncbi.nlm.nih.gov/LocusLink/), [PIR iProClass](http://pir.georgetown.edu/iproclass/), [UniProt](http://www.pir.uniprot.org/)  and [GOA](http://www.ebi.ac.uk/GOA/) records. - Gene Ontology can be found at [http://www.geneontology.org](http://www.geneontology.org/).  - Panther Database at <http://www.pantherdb.org/>  - NCI Thesaurus database at [**http://nciterms.nci.nih.gov/NCIBrowser/Dictionary.do**](http://nciterms.nci.nih.gov/NCIBrowser/Dictionary.do) |
|  |  | **Protein Domains** **-** Data extracted from [LocusLink](http://www.ncbi.nlm.nih.gov/LocusLink/), [PIR iProClass](http://pir.georgetown.edu/iproclass/), [UniProt](http://www.pir.uniprot.org/) and [InterPro](http://www.ebi.ac.uk/interpro/) records.  **-** Additional information can be found at websites for [Pfam](http://www.sanger.ac.uk/Software/Pfam/), [COG/KOG](http://www.ncbi.nlm.nih.gov/COG/new/), [Blocks](http://blocks.fhcrc.org/), [SMART](http://smart.embl-heidelberg.de/),  [PDB](http://www.rcsb.org/pdb/), [ProDom](http://protein.toulouse.inra.fr/prodom/current/html/home.php), [PROSITE](http://au.expasy.org/prosite/), [TIGRFAMs](http://www.tigr.org/TIGRFAMs/index.shtml), [PRINTS](http://www.bioinf.man.ac.uk/dbbrowser/PRINTS/) and [SCOP](http://scop.mrc-lmb.cam.ac.uk/scop/). |
|  |  | **Pathways:**  - KEGG Pathways, KEGG Reaction, KEGG Compound:  <http://www.genome.jp/kegg/> - CGAP BioCarta Pathways: <http://cgap.nci.nih.gov/Pathways/BioCarta_Pathways>    - Biocarta: <http://www.biocarta.com/genes/index.asp>   | The BioCarta Site, including all of its contents, such as text, images, and the HTML used to generate the pages, ("Materials"), are protected by patent, trademark and/or copyright under United States and/or foreign laws. Except as otherwise provided herein, you may not use, download, upload, copy, print, display, perform, reproduce, publish, license, post, transmit, or distribute any Materials from this Site in whole or in part, for any commercial purpose without the specific permission of BioCarta, Inc. You may use, download, upload, copy, print, display, perform, reproduce, publish, license, post, transmit, or distribute any Materials from this Site in whole or in part, for a public, noncommercial purpose with the specific permission of BioCarta, Inc. and/or acknowledgement of BioCarta and citation of the pathway number. BioCarta claims no ownership rights in the content placed on the Site by you or other registered users. We grant you a personal, non-exclusive, non-transferable license to access our web site and the information contained here. Our site may contain software tools or databases that allow you to search and retrieve information. You may not sell, rent, lease, lend, transfer, any services provided on this site and you may not assign or sublicense this license. | | --- |   **-** BBID: <http://bbid.grc.nia.nih.gov/> |
|  |  | **General Annotation** - Data extract from [LocusLink](http://www.ncbi.nlm.nih.gov/LocusLink/), [PIR iProClass](http://pir.georgetown.edu/iproclass/) and/or [UniProt](http://www.pir.uniprot.org/). |
|  |  | **Functional Categories** - Data extract from [LocusLink](http://www.ncbi.nlm.nih.gov/LocusLink/), [PIR iProClass](http://pir.georgetown.edu/iproclass/), [UniProt](http://www.pir.uniprot.org/) and/or [COG/KOG](http://www.ncbi.nlm.nih.gov/COG/new/). |
|  |  | **Protein Interactions**  - HIV Interactions: [LocusLink](http://www.ncbi.nlm.nih.gov/LocusLink/) and <http://www.ncbi.nlm.nih.gov/RefSeq/HIVInteractions/> - NCICB caPathway:  [http://ncicb.nci.nih.gov](http://ncicb.nci.nih.gov/) - Bind: <http://www.blueprint.org/bind/bind.php>- Mint: [http://mint.bio.uniroma2.it/mint/](http://http:/mint.bio.uniroma2.it/mint/) - DIP: [http://dip.doe-mbi.ucla.edu](http://dip.doe-mbi.ucla.edu/)  - HPRD <http://www.hprd.org/> |
|  |  | **Literature** - PubMed ID:  Data extract from [LocusLink](http://www.ncbi.nlm.nih.gov/LocusLink/), [PIR iProClass](http://pir.georgetown.edu/iproclass/) and/or [UniProt](http://www.pir.uniprot.org/). - GeneRIF: Extracted from [Entrez Gene](http://www.ncbi.nlm.nih.gov/Entrez/) |
|  |  | **Disease Association -** Genetic Association Database: <http://geneticassociationdb.nih.gov/> - OMIM Phenotype: [LocusLink](http://www.ncbi.nlm.nih.gov/LocusLink/) and [OMIM](http://www.ncbi.nlm.nih.gov/entrez/query.fcgi?db=OMIM) |
|  |  | **Gene Tissue Expression** - GNF Microarray: <http://wombat.gnf.org/index.html> - CGAP SAGE: <http://cgap.nci.nih.gov/SAGE> - CGAP Tissue EST: <http://cgap.nci.nih.gov/Tissues> - NCBI Unigene EST Profile: <ftp://ftp.ncbi.nih.gov/repository/UniGene/Homo_sapiens/> |
|  |  | **Other Data Sources** **-** HomoloGene: <http://www.ncbi.nlm.nih.gov/entrez/query.fcgi?db=homologene> |

**Contributions of Public Data Resources to DAVID Knowledge Base Construction**

**Listed by independent databases:**

1) Entrez Gene database: <http://www.ncbi.nlm.nih.gov/Entrez/>

2) Protein Information Resource (PIR) <http://pir.georgetown.edu/>

3) Uniprot/Swiss-prot: <http://www.pir.uniprot.org/>

4) Gene Ontology database: [http://www.geneontology.org](http://www.geneontology.org/)

5) NetAffy database: <http://www.affymetrix.com/analysis/index.affx>

6) TIGER database: <http://www.tigr.org/tdb/>

7) Interpro database: <http://www.ebi.ac.uk/interpro/>

8) Pfam database: <http://www.sanger.ac.uk/Software/Pfam/>

9) COG/KOG databse: <http://www.ncbi.nlm.nih.gov/COG/new/>

10) Blocks database: <http://blocks.fhcrc.org/>

11) SMART database: <http://smart.embl-heidelberg.de/>

12) PDB database: <http://www.rcsb.org/pdb/home/home.do>

13) PDOM database: <http://prodom.prabi.fr/prodom/current/html/home.php>

14) Prosite database: <http://au.expasy.org/prosite/>

15) Tigr fams database: <http://www.tigr.org/TIGRFAMs/index.shtml>

16) PRINTS database: <http://www.bioinf.man.ac.uk/dbbrowser/PRINTS/>

17) SCOP database: <http://scop.mrc-lmb.cam.ac.uk/scop/>

18) KEGG database: <http://www.genome.jp/kegg/>

19) CGAP Pathway database: <http://cgap.nci.nih.gov/Pathways/BioCarta_Pathways>

20) BIDD database: <http://bbid.grc.nia.nih.gov/>

21) iProClass database: <http://pir.georgetown.edu/iproclass/>

22) HIV Interaction database: <http://www.ncbi.nlm.nih.gov/RefSeq/HIVInteractions/>

23) BIND database: <http://www.blueprint.org/bind/bind.php>

24) MINT database: <http://mint.bio.uniroma2.it/mint/>

25) DIP database: <http://dip.doe-mbi.ucla.edu/>

26) Genetic Assoication database: <http://geneticassociationdb.nih.gov/>

27) OMIM database: <http://www.ncbi.nlm.nih.gov/entrez/query.fcgi?db=OMIM>

28) Homologene database: <http://www.ncbi.nlm.nih.gov/entrez/query.fcgi?db=homologene>

29) GNF database: <http://wombat.gnf.org/index.html>

30) CGAP Genie database: <http://cgap.nci.nih.gov/SAGE>

31) CGAP Tissue database: <http://cgap.nci.nih.gov/Tissues>

32) NCBI Unigene database: <http://www.ncbi.nlm.nih.gov/entrez/query.fcgi?db=unigene>

33) NCBI Pubmed database: <http://www.ncbi.nlm.nih.gov/entrez/query.fcgi?DB=pubmed>

34) NCBI Generif database: <http://www.ncbi.nlm.nih.gov/projects/GeneRIF/GeneRIFhelp.html>

35) GOA database: <http://www.ebi.ac.uk/GOA/>

36) Panther database: <http://www.pantherdb.org/>

37) NCI Therausus database: <http://nciterms.nci.nih.gov/NCIBrowser/Dictionary.do>

38) HPRD <http://www.hprd.org/>
