# Supplementary material for: DAVID Knowledgebase: a gene-centered database integrating heterogeneous gene annotation resources to facilitate high-throughput gene functional analysis
Source: BMC Bioinformatics. 2007 Nov 2;8:426. doi: 10.1186/1471-2105-8-426 (PMC2186358; doi:10.1186/1471-2105-8-426)
Supplement: Additional file 4 — An outline of the update procedure for the DAVID Knowledgebase. [file 1471-2105-8-426-S4.doc]

**DAVID Knowledgebase Update Procedure**


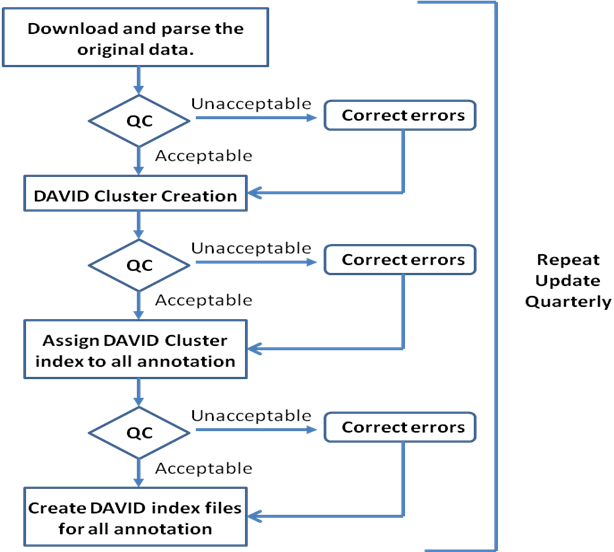


The update process begins with the download and parsing of the various data sources (additional file 3) using in-house parsers. The process is closely monitored and any problems are recorded and corrected. In addition, a quality control evaluation is performed on the resulting files in order to verify integrity. This evaluation includes size and content comparisons with human curation where necessary. Once the integrity of the parsed files is verified, the DAVID Cluster creation begins (figure 1b). Upon completion, the DAVID Quality Control Pipeline is invoked to ensure the integrity of the clusters and to identify any incorrectly assigned annotation (additional file 2). A unique index is assigned to each cluster after QC is complete and the indexes are then merged with all other appropriate annotation. Another QC step is performed to verify correct assignment and the final annotation specific DAVID index files are produced as a two-column tab-delimited text file (figure 2b). The goal for the knowledgebase update process is set to occur quarterly. While this schedule may not completely coincide with the updates of all of the databases involved in the update process, the redundant and complementary nature of the various annotation sources included in the knowledgebase will allow for up-to-date high-throughput gene functional annotation for which the knowledgebase is intended.
