# Supplementary material for: DAVID Knowledgebase: a gene-centered database integrating heterogeneous gene annotation resources to facilitate high-throughput gene functional analysis
Source: BMC Bioinformatics. 2007 Nov 2;8:426. doi: 10.1186/1471-2105-8-426 (PMC2186358; doi:10.1186/1471-2105-8-426)
Supplement: Additional file 5 — The performance of querying the DAVID Knowledgebase through the web interface with different size gene lists. [file 1471-2105-8-426-S5.doc]

**DAVID Knowledgebase Interface Query Performance**

In order to measure the scalability of the DAVID Knowledgebase Interface, we measured the time(in seconds) taken to query both Gene Ontology Biological Process terms alone as well as all Gene Ontology terms for different size lists of genes as outlined below. To avoid the variation of bandwidth and browser capability from user to user, the query time is measured based on the server-side time cost only. The numbers of unique gene-term pairs, as well as the number of genes in the list, are used for the comparison and charted against time(s).

1. **Demolist 1** – A list of 164 Affymetrix IDs found to be upregulated in CD4+/CD62L- T cells relative to CD4+/CD62L+ T cells in a microarray experiment(labeled as Demolist 1 on the DAVID web site) [*Hengel RL, et. al., J Immunol 2003*].
2. **Demolist2** – A list of 403 Affymetrix IDs derived from a HIV microarray study (labeled as Demolist 2 on the DAVID web site) [23]
3. **Hu6800** – A list of 7129 Affymetrix IDs found on the Affymetrix Hu6800 microarray.

Result Table:

| **Gene List** | **Annotation** | **#Genes in List** | **#Unique Gene-Term Pairs** | **Time(seconds)** |
| --- | --- | --- | --- | --- |
| Demolist1 | GO Biological Process | 164 | 2775 | 0.01 |
| Demolist1 | All Go Terms | 164 | 5022 | 0.019 |
| Demolist2 | GO Biological Process | 403 | 8867 | 0.026 |
| Demolist2 | All Go Terms | 403 | 14848 | 0.043 |
| Hu6800 | GO Biological Process | 7129 | 162076 | 0.516 |
| Hu6800 | All Go Terms | 7129 | 290487 | 0.912 |

**
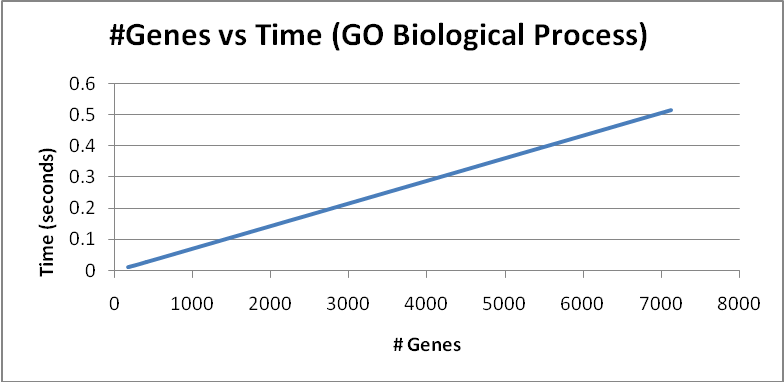
**

**Figure 1. The query time for corresponding GO terms of BP increases linearly for increasing sizes of gene lists.**

**
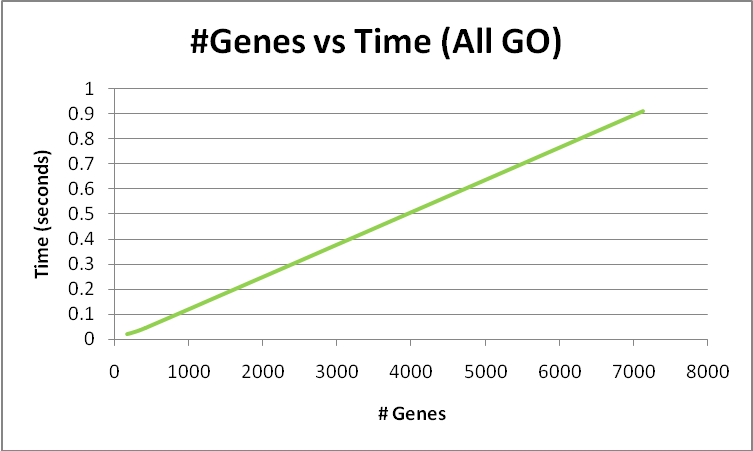
**

**Figure 2. The query time for corresponding GO terms in all levels increases linearly for increasing sizes of gene lists.**


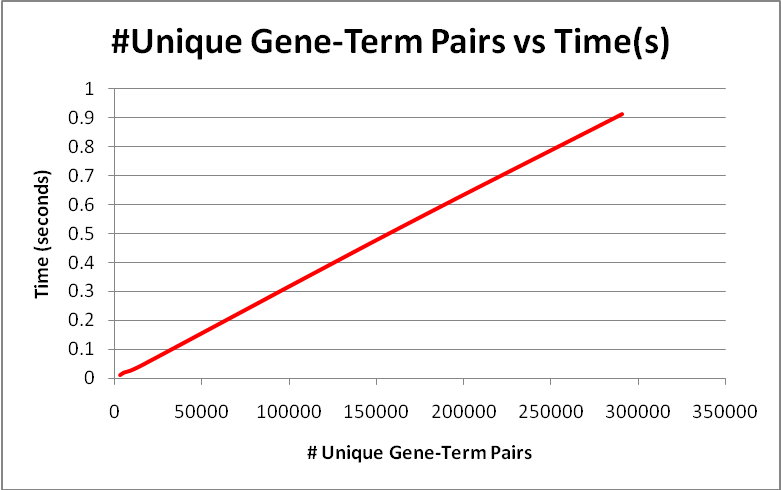


**Figure 3. The query time is increased linearly as the # unique gene-term pairs increases.**
