# Supplementary material for: DAVID Knowledgebase: a gene-centered database integrating heterogeneous gene annotation resources to facilitate high-throughput gene functional analysis
Source: BMC Bioinformatics. 2007 Nov 2;8:426. doi: 10.1186/1471-2105-8-426 (PMC2186358; doi:10.1186/1471-2105-8-426)
Supplement: Additional file 7 — The comparisons of querying GO terms with DAVID, SOURCE, RESOURCER, IDConverter, BioMart and UCSC Gene Sorter for the demolist 2 [26]. [file 1471-2105-8-426-S7.doc]

**Comparison of the DAVID Knowledgebase Interface with other high-throughput database interfaces.**

A list of 403 Affymetrix IDs derived from a HIV microarray study (labeled as demolist 2 on the DAVID web site) [23] is used to query the DAVID Knowledgebase and the other databases respectively in order to obtain associated biological annotations.

The criteria for the comparison are the same as that used in the comparison presented in the manuscript between the DAVID knowledgebase and NCBI’s Entrez Gene.

1. Does the interface accept Affymetrix probeset ids directly? If not, ids are converted to Entrez Gene ids.
2. Does the interface accept id input in batch?
3. Does the interface provide a tabular download of the results?
4. How many of the original gene ids are mapped to the chosen annotation (Gene Ontology)?
5. How many unique gene-term pairs are returned by the interface?

| **INTERFACE** | **Accept Affymetrix** | **Batch Input** | **Tabular Download** | **#Genes Mapped** | **#Unique Gene-Term Pairs** |
| --- | --- | --- | --- | --- | --- |
| DAVID | Yes | Yes | Yes | 371 | 14496* |
| BioMart | Yes | Yes | Yes | 346 | 3443 |
| UCSC GeneSorter | Yes | Yes | Yes | 234 | 2401 |
| IDConverter | Yes | No* | Yes | 331 | 2837 |
| SOURCE | No | Yes | Yes | 330 | 2364 |
| RESOURCER | No | Yes | Yes | 142 | 1812 |

* The improved number is due to the integration of multiple GO sources by the DAVID Knowledgebase, as well as to the incomplete GO levels collected in other databases.
